# Designing Ecofriendly Bionanocomposite Assembly with Improved Antimicrobial and Potent on-site Zika Virus Vector Larvicidal Activities with its Mode of Action

**DOI:** 10.1038/s41598-017-15537-9

**Published:** 2017-11-14

**Authors:** Pramod C. Mane, Ravindra D. Chaudhari, Manish D. Shinde, Deepali D. Kadam, Chung Kil Song, Dinesh P. Amalnerkar, Haiwon Lee

**Affiliations:** 1 P. G. Department of Zoology and Research Centre, Shri Shiv Chhatrapati College of Arts, Commerce and Science, Junnar, Pune, 410 502 India; 2 Centre for Materials for Electronics Technology, Panchwati, Off Pashan Road, Pune, 411 008 India; 3 Institute of Nano Science and Technology, Hanyang University, Seoul, 04763 South Korea; 4 School of Mechanical Engineering, Sungkyunkwan University, Suwon, 440 746 South Korea; 5 Department of Chemistry, Hanyang University, Seoul, 04763 South Korea

## Abstract

Dialyzed natural polymer, fibroin, from *Bombyx mori* was used to synthesize biocompatible silver and gold nanoparticles *in-situ* in dispersion form. The films of pure fibroin (PF), fibroin-silver nanocomposite (FSNC) and fibroin-gold nanocomposite (FGNC) were fabricated by drop casting method. The characterization of the resultant dispersion and films was performed by visual color change, UV-Vis spectroscopy and atomic force microscopy. The dispersions of PF, FSNC and FGNC were tested for antibacterial activity against *E. coli* NCIM 2065, *S. aureus* NCIM 5021, *K. pneumoniae* NCIM 2957, *P. aeruginosa* ATCC 9027 and antifungal activity against *A. fumigatus* NCIM 902. FSNC dispersion exhibited an effective antimicrobial action against all the tested microbes as compared to FGNC dispersion. The mechanism of action for FSNC and FGNC against these microorganisms is proposed. Additionally, the larvicidal activity of the films was investigated against the larvae of *Aedes aegypti*. The films of FSNC exhibited 100% mortality while the films of FGNC revealed 86–98% mortality against all the larval instars and pupae of *A. aegypti*. The phytotoxicity study of the nanocomposite films was also carried out to confirm the reusability of water. This is first noble metal nanocomposite based report on larvicidal activity of zika virus vector.

## Introduction

Of late, biomaterials have been playing a vital role in medical devices and regenerative medicines^1^. From the standpoint of biomedical applications, there has been growing concern in the development of innovative, clean and green health care products particularly because the development of antibiotic resistant microorganisms has imposed a myriad of new challenges^2^. As an obvious consequence of antimicrobial resistance, several researchers are constantly engaged in developing new antimicrobial agents/drugs^3–5^. Traditionally, gold and silver have been used in the medical field since ancient times, especially; silver has been used in antimicrobial applications such as burn treatment^6^. It has been also stated that silver can prevent HIV from binding to host cell^7^. These non-essential metals in chemical biology possess toxicity towards bacteria at exceptionally low concentrations^8^. Through the oxidation process, metals can catalyze site specific damage to cellular proteins leading to loss of catalytic activity and triggering an active process of protein degradation. However, there are some limitations in using metal ions and salts as antimicrobial agents and the probable reason includes the interfering effects of salts. Such limitations can be circumvented by using the metals in nano-structured form^9^ since nanoparticulate systems are emerging as new antimicrobial path which takes the advantage of bactericidal and fungicidal tendency of highly active metallic nanoparticles^10^. Besides, biomaterials (viz. cotton, chitin, chitosan, alloskin, pigskin etc.) pre-treated with metal nanoparticles have been widely explored for wound dressing. But as these nanocomposite materials hold low antimicrobial activity, allergenicity, toxic effects and poor adhesiveness, they might not lead to extensive therapeutic applications^11^.

Likewise, another challenging issue is the insects control and their increased resistance to many of the existing insecticides. This envisages the development of new insect control tools^12^. In particular, the major vector for spreading of zika-flavivirus, malaria, dengue, yellow fever, filariasis, schistosomiasis, Japanese encephalitis and chikungunya are mosquitoes including *Aedes aegypti*. The survey conducted by WHO revealed that the number of deaths and infected cases of malaria were found to be increased continously^13,14^. According to a recent study, it is projected that several billions of people are at risk of dengue infection in 128 countries worldwide^15^. Earlier, mosquito-spread zika virus which causes dengue like symptoms was mostly detected in Asia and Africa. It has been also reported that the main vectors of this virus are more infamous *Aedes aegypti* and less known anthropophilic *Aedes albopictus* ^16^. In Africa and Asia, zika virus has been neglected for decades because it generally results in moderate or clinically asymptomatic infections^17^. However, after 2015 outbreak in Brazil, zika virus got an attention as it has impact on human health leading to neurological injuries and adverse fetal outcomes^18^. From September 22, 2015 to March 19, 2016, there were 58,838 cases of zika virus infection in Colombia^19^. Before the outbreak in Latin America, zika virus was not linked to any deaths. Such cases including reports of Brazil Ministry of Health related to three deaths associated with zika virus infection calls attention to generate evidence based guidelines for management of zika as well as possible vectors like *Aedes aegypti* ^20–22^. Curiously, the use of nanomaterials in the management of zika by controlling the vector growth has not been reported up till now.

Synthesis of application-oriented nanoparticles and systems using routes which are green, facile and cost-effective has been the cradle of recent research. In this context, biological synthesis of nanoparticles can play a vital role. Almost all types of biological materials including microorganisms, various parts of plants, algae etc. have been exploited for the synthesis of nanoparticles^23–26^. Although the usage of nanoparticles for killing microorganisms, insect control, crop protection etc. is well recognized, the nanoparticle systems with immobilized nanoparticles are of significant interest from the ecological viewpoint. Immobilization of nanoparticles on a surface promotes its reuse and also reduces environmental risks as surface immobilization minimizes/controls leaching of nanoparticles^27^. Silk based biomaterials have been found to be highly biocompatible with various cell types as they can provide good support for cell adhesion as well as proliferation and also do not cause any major cell toxicity^28–30^. Another significance of using silk biopolymers is, they degrade slowly as compared to other natural polymers and the degradation by-products are harmless to human body^31^. Silk fibroin based materials were explored in the past decades as healthy foods, in biosensors, drug delivery systems and used in many fields revolving around biology, medicine, materials and chemical engineering^32–37^. Specifically, the fibroin obtained from *B. mori* cocoons has synergistic mechanical and biological properties viz. non-toxicity, biodegradability and biocompatibility and its composite with nanosilver holds a great promise for antimicrobial activity^37^.

Keeping in mind the above aspects, we generated silver and gold nanoparticles based silk fibroin nanocomposite dispersions and films by using clean, green and facile method with promising antimicrobial as well as larvicidal (*A. aegypti*) activities. Also, we report the novel protocol for the effective surface immobilization of metallic nanoparticles with retention of their antimicrobial and larvicidal actions. Herein, we further conjecture that the use of such protocol increases the long-term usage of nanoparticles and also protects the aquatic ecosystem with controlled metal ion release. The surface immobilized nanoparticles evince greater bactericidal activity than the colloidal ones and additionally promote reuse. It has been well established that the bactericidal activity of nanoparticles escalates when they are immobilized (primarily due to contact-mode killing mechanism) in comparison to free nanoparticles in dispersion which are susceptible to spontaneous aggregation limiting bactericidal efficacy^38,39^.

## Results and Discussion

### Physico-chemical characterization of FSNC and FGNC dispersions and films

**Figure 1:**
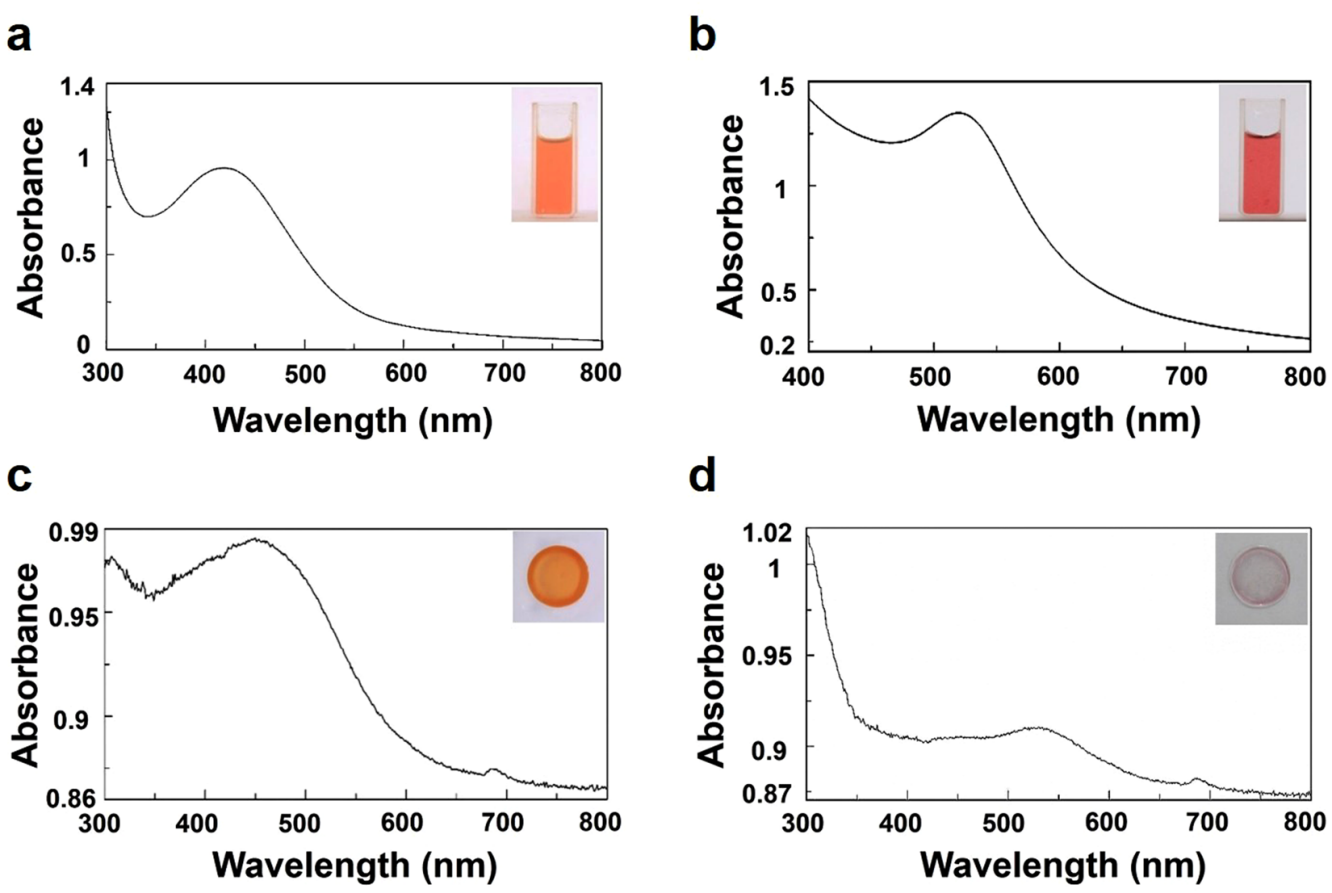
UV-visible spectra of (**a**) silver nanoparticles dispersion, (**b**) gold nanoparticles dispersion, (**c**) film of silver nanoparticles and (**d**) film of gold nanoparticles.

**Figure 2:**
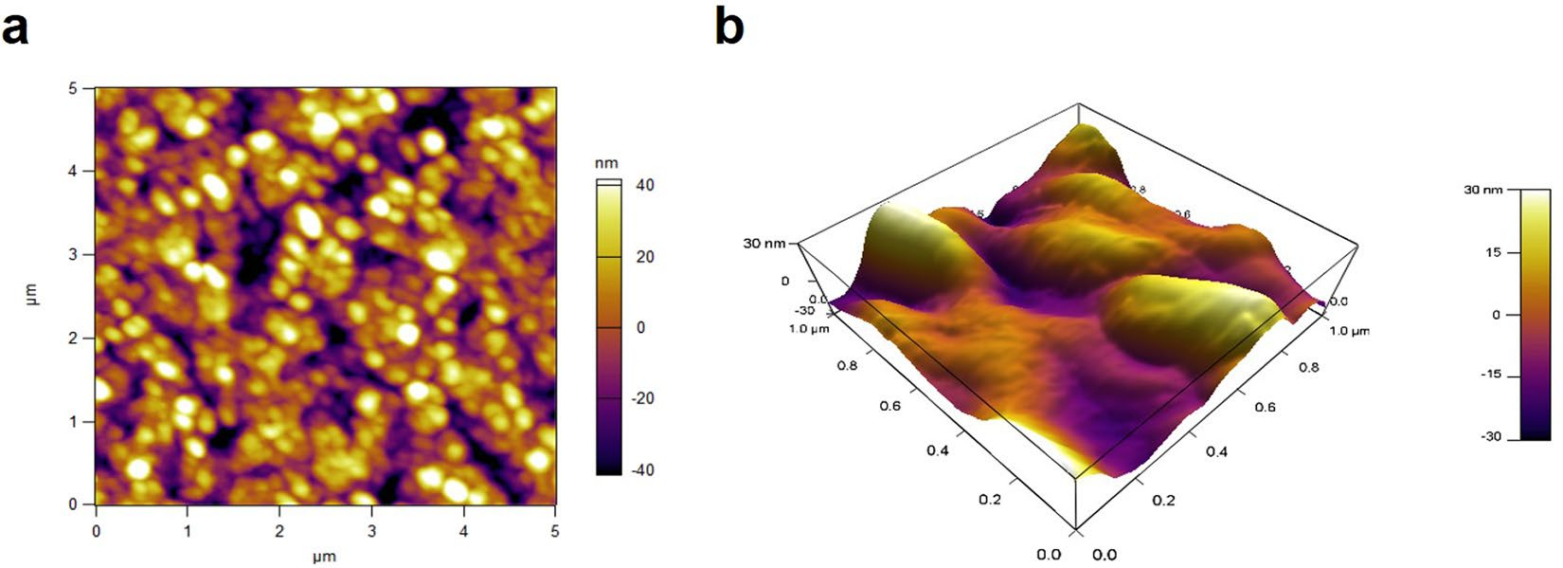
AFM images of silk fibroin films containing silver nanoparticles (**a**) 2D and (**b**) 3D view.

**Figure 3:**
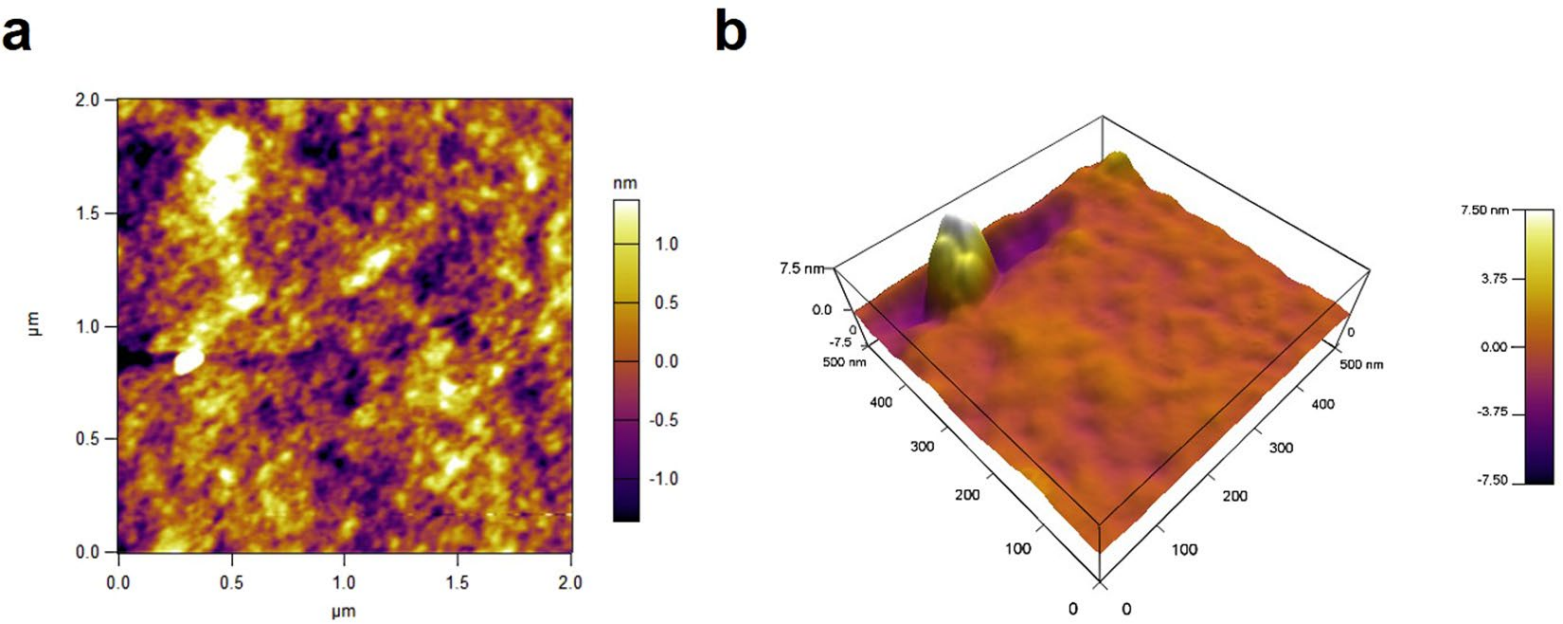
AFM images of silk fibroin films containing gold nanoparticles (**a**) 2D and (**b**) 3D view.

The UV-visible spectra of FSNC and FGNC dispersions are shown in Fig. 1a and b, respectively. The UV-visible spectra in diffuse reflectance (DRS) mode of the silk films with silver and gold nanocomposites are shown in Fig. 1c and d, respectively. The silk fibroin dispersion and film corresponding to silver nanoparticles (Fig. 1a and c) reveal a broad surface plasmon resonance (SPR) peak around 350–550 nm. However, FSNC dispersion exhibited relatively narrower peak centered around 420 nm as compared to FSNC film which has a peak centered around 460 nm. The characteristic peak for spherical silver nanoparticles mainly lies in the range of 420 nm^40^. The gold nanoparticles captured in silk solution as well as in film exhibit a surface plasmon resonance (SPR) peak at 520 nm, which is a characteristic peak for gold nanoparticles as displayed in Fig. 1b and d, respectively^41^. Analogous to FSNC samples, the FGNC samples also demonstrate the relative enhancement in the peak broadness for the film form in comparison to the dispersion. The broadness of the peak towards the higher wavelength side may be due to capping environment (proteins) and/or due to wide-ranging particle size distribution. It has been reported that the position and broadness of SPR peak of gold and silver nanoparticles depend on many factors such as particle size, size distribution, shape, surrounding dielectric environment, type of solvent etc^42^. Atomic force microscopy (AFM) images of silver nanoparticles-SF film are presented in Fig. 2. The AFM image at low magnification (Fig. 2a) shows the formation of spherical and elongated silver nanoparticles in the form of bunches and distributed throughout the SF matrix. Higher magnification, 3-dimensional (3D) view (Fig. 2b) reveals that each nanoparticulate bunch is made up of smaller particles which are nanometers in dimensions. AFM images of gold nanoparticles-SF film are displayed in Fig. 3. The low magnification image discloses the formation of very smaller nanoparticles which are evenly distributed within the SF matrix. Occasionally, very big micron size particles are also observed above the surface of the film which do not seem to be formed within the polymer matrix.

These could be the dust particles which might have accidently got deposited over the surface during any stage of processing. The higher magnification 3D view images (Fig. 3b) reveal that the nanoparticles have very small particle size distribution and their size is confined to the range of 20–30 nm.

### Antimicrobial Activities of FSNC and FGNC films

**Figure 4:**
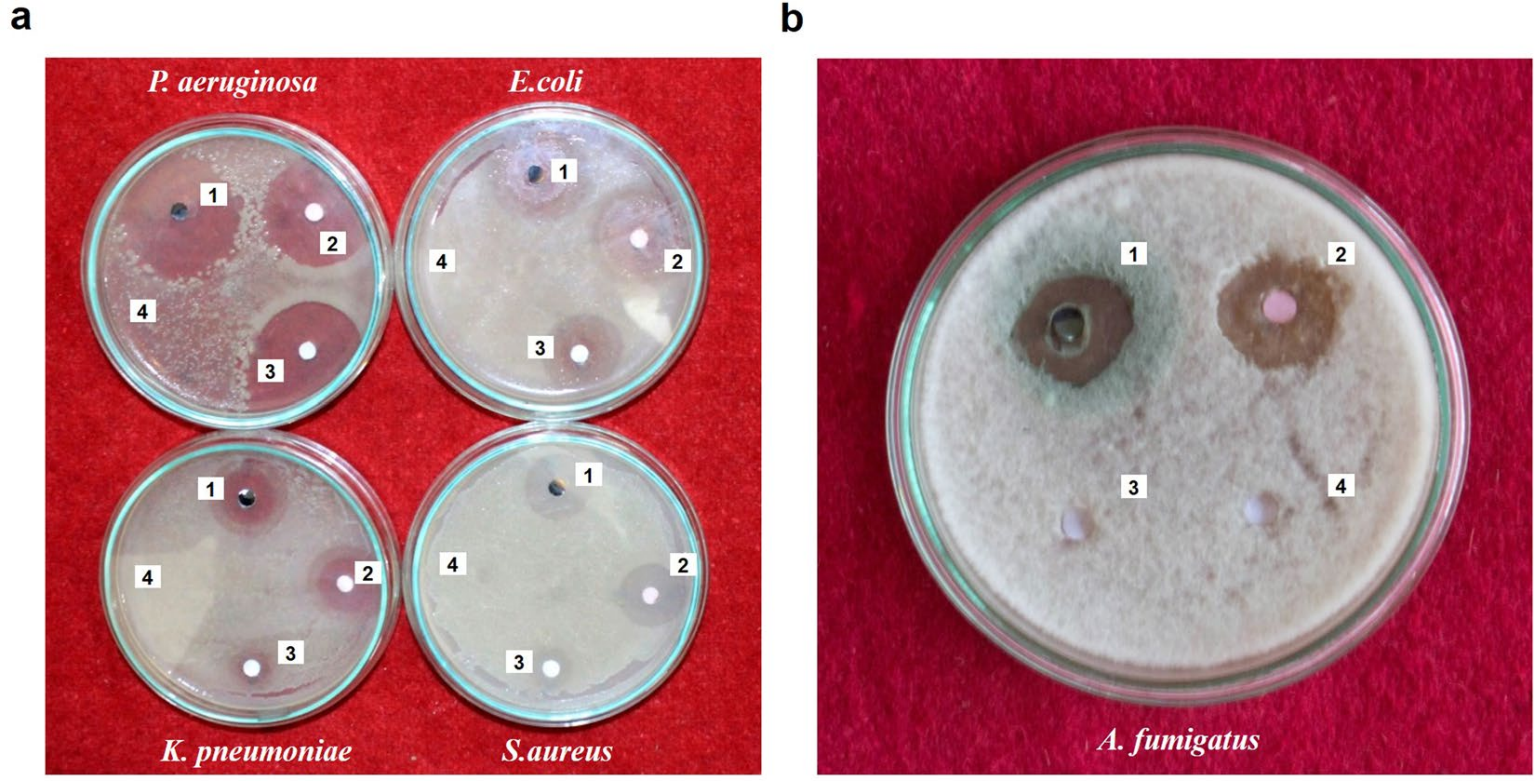
(**a**) Antibacterial activity of (1) FSNC, (2) FGNC (3) Ciprofloxacin and (4) PF and (**b**) Antifungal activity of (1) FSNC, (2) FGNC (3) Clotrimazole and (4) PF.

In the present antimicrobial study, green nanocomposite materials consisting of silver and gold nanoparticles have been used. The antibacterial activity of FSNC and FGNC dispersion was verified by observing a clear zone of inhibition. No zone of inhibition was found in the vehicle control well which suggested that the antimicrobial activity was specifically due to the nanocomposites. All the selected four bacterial strains and one fungal strain demonstrated clear zone of inhibition when treated with nanocomposite dispersion (Fig. 4a and b). Both FSNC and FGNC exhibited maximum zone of inhibition against *P. aeruginosa* (4.2 ± 0.25 cm and 3.1 ± 0.05 cm, respectively) while *S. aureus* exhibited comparatively less zone of inhibition as illustrated in Table 1(next section). Closely following the literature, it was noted that compared to Gram-positive bacteria, the growth of Gram-negative bacteria is inhibited at higher concentrations of nanoparticles^43^. It is also reported that the higher susceptibility of Gram-negative bacteria could be related to the differences in the structure of the cell wall, cell physiology, metabolism and composition of the cell wall^44^. In the present research work, fibroin based nanocomposites exhibited pronounced antibacterial activity against Gram-negative bacteria as well. In particular, we compared the antibacterial activity of FSNC and FGNC with ciprofloxacin and antifungal activity with clotrimazole. Ciprofloxacin is effectively administered against various Gram-positive and Gram-negative bacteria though there are recent signals of upsurgence in its bacterial resistance in clinical practice^45^. Clotrimazole is an antifungal medication primarily used to treat vaginal yeast infections, oral thrush and various types of ringworms^46^. Our results (Fig. 4a) disclosed that FSNC and FGNC exhibit better antimicrobial activity than the standard antibiotic used namely ciprofloxacin which exhibits good antibacterial activity against *P. aeruginosa* and *E. coli* but relatively less activity for *S. aureus* and *K. pneumoniae*. Less propensity towards bacterial resistance would be an additional advantage for our nanocomposites. Interestingly, we observed that standard antifungal drug clotrimazole does not exhibit any antifungal activity against *A. fumigatus* while FSNC and FGNC have shown substantial antifungal activities (Fig. 4b).

**Table 1:** Zone of inhibition and MIC of FSNC and FGNC.

Name of the Organism	Zone of Inhibition (cm)			MBC/MFC (µg/ml)	
	FSNC	FGNC	Control	FSNC	FGNC
*K. pneumoniae*	3.0 ± 0.11	2.6 ± 0.25	1.3 ± 0.05	2	6
*E. coli*	3.4 ± 0.25	2.9 ± 0.15	2.6 ± 0.25	10	10
*P. aeruginosa*	4.2 ± 0.25	3.1 ± 0.05	3.4 ± 0.26	10	10
*S. aureus*	2.6 ± 0.11	2.2 ± 0.11	1.2 ± 0.20	8	10
*A. fumigatus*	3.3 ± 0.26	1.2 ± 1.15	00	2	10

### Evaluation of antimicrobial effectiveness using minimum inhibitory concentration method

The antimicrobial activity of nanoparticles was ascertained by evaluating MIC, MBC and MFC. The antimicrobial effectiveness was determined against the microbial concentrations of 10^6^ CFU/ml with different concentrations of FSNC and FGNC namely, 2, 4, 6, 8, 10 μg/ml. It was noticed that, as the concentration of nanoparticles increases, the final microbial concentration decreases. MIC (the minimum concentration at which a visual inhibition of growth occurs) of FSNC against *K. pneumoniae, E. coli, P. aeruginosa, S. aureus* and *A. fumigatu*s was found to be 2, 10, 10, 8 and 2 μg/mL, respectively. In case of FGNC, the MIC values were 6, 10, 10, 10 and 10 μg/ml against *K. pneumoniae, E. coli, P. aeruginosa, S. aureus* and *A. fumigatus*, respectively. The corresponding MBC and MFC values are as shown in Table 1.

### Effects of FSNC and FGNC on bacterial growth with the mode of action

**Figure 5:**
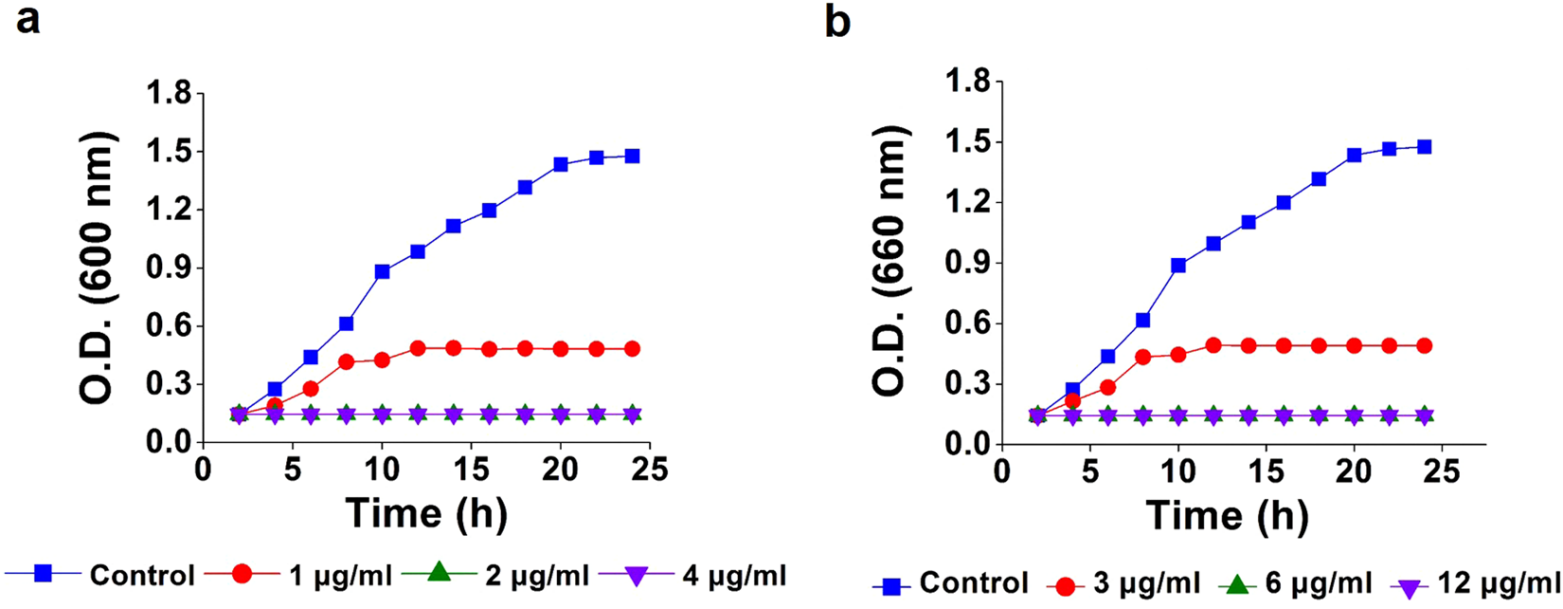
Effects of (**a**) FSNC and (**b**) FGNC on growth curves of *K. pneumonia* at various concentrations of nanocomposites.

The typical growth curves of bacterial cells (e.g. *K.pneumonia*) treated with FSNC and FGNC (Fig. 5) indicated that the nanocomposite could inhibit the growth and reproduction of bacterial cells. The growth of bacterial cells *K. pneumoniae* treated with 2 and 4 μg/ml of FSNC was inhibited as specified in Fig. 5a, while the growth of bacterial cells *K. pneumoniae* treated with FGNC was inhibited at 6 and 12 μg/ml as shown in Fig. 5b. After three hours, almost all treated bacterial cells were dead. MIC of both FSNC and FGNC was also found to be lower than that of the control group. These findings indicate that the antibacterial activity of 1 μg/ml of FSNC and 3 μg/ml of FGNC could inhibit the growth of *K. pneumoniae* but not enough to reduce the speed of reproduction of the bacterial cells. There are some reports about the DNA damage in *E. coli*, catalyzed by Fe-mediated Fenton Chemistry^47^. Metal nanoparticles like silver, gold possess the ability to cause structural changes in the cell membrane, DNA damage and subsequently the cell death. They also form “pits” on cell surface increasing the accumulation of Ag and Au nanoparticles in the cell wall^48–50^. It was established that FSNC and FGNC could enhance protein reducing sugar, DNA and RNA leakage by increasing the membrane permeability of the bacteria considered. Initially, protein leakage from the cell membranes of *K. pneumoniae* cells treated with nanoparticles was almost the same as that from cells in the control group. After 6 hours of incubation, protein leakage from cells treated with nanoparticles considerably increased; however, there was no change in the amount of protein leakage from the cells in the control group (Table 2). Study on leakage of UV260 and UV280 absorbing material was monitored over the period of 90 minutes and the results are presented in Table 3. The absorbance at 280 nm was increased in 15 minutes when compared to the absorbance at 260 nm for FSNC and FGNC. These results suggest that the nanoparticles can alter the membrane permeability resulting in the leakage of the UV260 and UV280 absorbing material. Leakage of the intracellular material leads to cell death, which seems to be one of the important bactericidal actions^51^. It has also been recorded that there can be release of metal ions by nanoparticles which can interact with the thiol groups of many vital enzymes and inactivate them^52,53^. The bacterial cells in contact with gold and silver nanocomposites consume gold and silver ions, which in turn, inhibit several cell functions and damage the cells. Gold and silver are soft acids and hence have natural tendency to react with base^54^. In this case, soft acids react with soft bases as the cells are mainly composed of sulphur and phosphorus which are soft bases. Another mechanism is that the DNA has sulphur and phosphorus as major components and hence nanoparticle can act on these soft bases and destroy the DNA leading to cell death. Presumably, in the present case, the interaction of Ag and Au nanoparticles with sulphur and phosphorus of DNA can lead to problems in the DNA replication and hence can terminate the microbes^55^.

**Table 2:** Cell membrane Leakage of FSNC and FGNC.

Time of Exposure	Parameters											
	Protein			Reducing Sugar			DNA			RNA		
	Control	FSNC	FGNC	Control	FSNC	FGNC	Control	FSNC	FGNC	Control	FSNC	FGNC
0 Hr	4.50	4.50	4.50	2.71	2.71	2.71	0.00	0.00	0.00	0.00	0.00	0.00
5 Mins	4.50	7.74	5.98	2.71	12.86	10.64	0.00	0.00	0.00	0.00	0.00	0.00
6 Hr	4.52	22.95	21.73	2.71	107.5	99.85	0.00	0.56	0.48	0.00	0.08	0.07

**Table 3:** Heipieper Method of FSNC and FGNC.

Time (min.)	Absorbance at					
	UV_260_ nm			UV_280_ nm		
	Control	FSNC	FGNC	Control	FSNC	FGNC
15	0.04	0.19	0.18	0.05	0.32	0.26
30	0.16	0.21	0.18	0.21	0.35	0.26
45	0.16	0.34	0.32	0.20	0.41	0.39
60	0.17	0.34	0.32	0.21	0.41	0.39
75	0.17	0.36	0.32	0.21	0.43	0.42
90	0.16	0.37	0.34	0.21	0.45	0.45

### Mosquito Larvicidal activity

**Figure 6:**
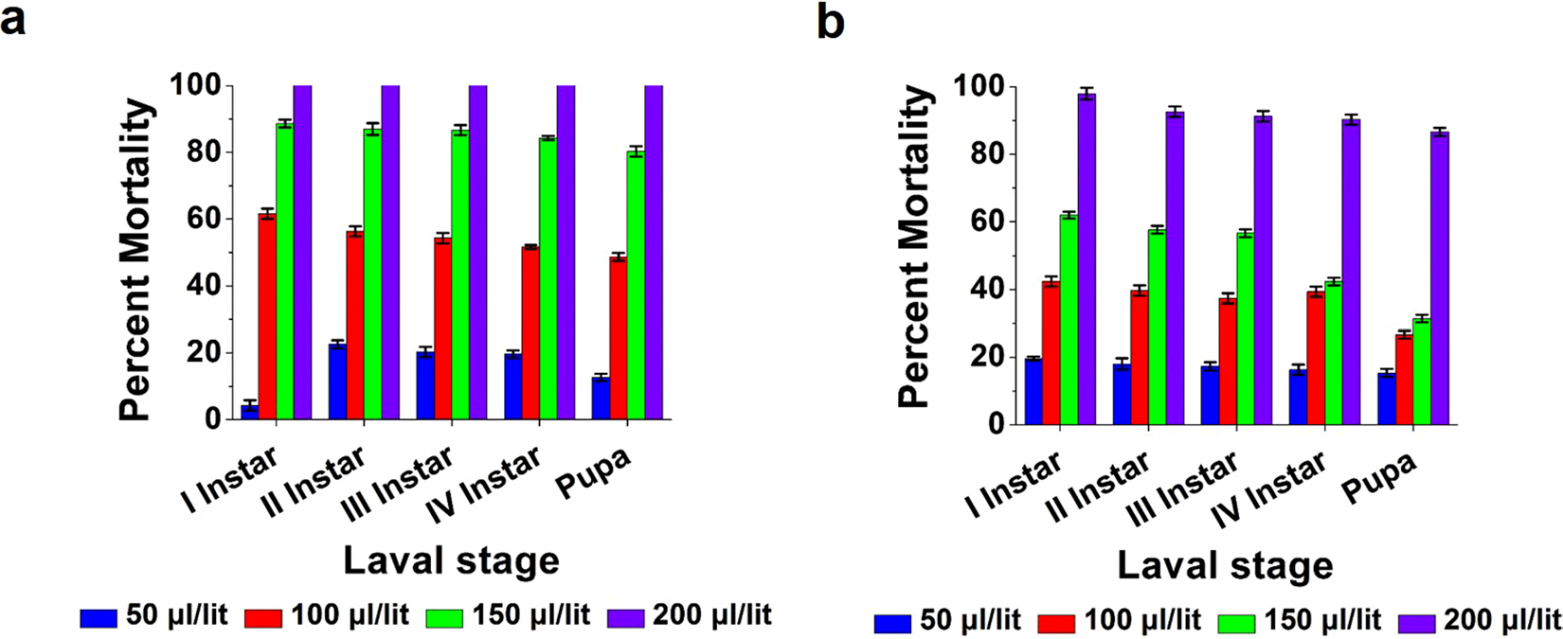
Larval and pupal toxicity effects of (**a**) FSNC and (**b**) FGNC against *Aedes aegypti*.

**Figure 7:**
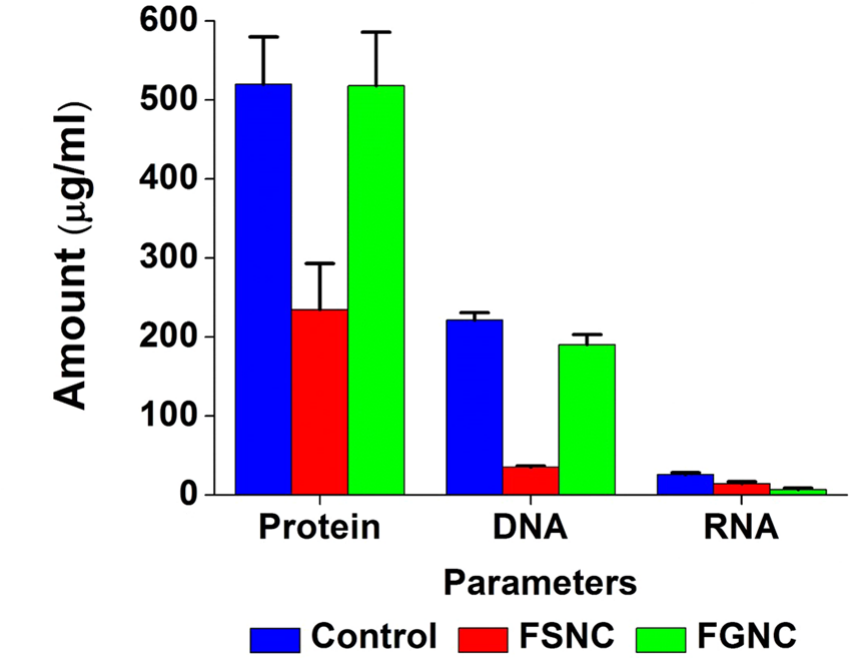
Effects of PF, FSNC and FGNC films on biochemical parameters of *Aedes aegypti* larvae.

Insect vector control is an important aspect for the comfort of human beings. Unfortunately, due to the lack of development of eco-friendly biopesticides/insecticides and emergence of resistance, especially, in vector mosquitoes to the conventional synthetic insecticides is facing a serious threat worldwide. In the present study, films of PF, FSNC and FGNC corresponding to various quantities viz., 50, 100, 150 and 200 μl/lit were tested against 1^st^, 2^nd^, 3^rd^, 4^th^ instars and pupae of *A. aegypti*. Such bionanocomposite films were found to be stable and were subjected to dehydration by passing them through various grades of ethanol. It was also observed that once the fibroin gets dried or sets into a gel, it does not get dissolved in water. It has been reported that metallic nanoparticles generally display lower acute toxicity than their respective metal ions which, in turn, depends upon their concentration, size, biodistribution, bioavailability and medium chemistry^38^. Besides, it is anticipated that the surface immobilization of metallic nanoparticles in biopolymeric matrix with the controlled metal-release kinetics can minimize their entry in aquatic and other ecosystems. Considering all such beneficial ecotoxicological aspects, larvicidal bioassay was conducted by using the nanocomposite films instead of their dispersions. From the relevant results, we noted that the PF did not exhibit any mortality in all the tested larval and pupal stages of *A. aegypti*. Percent mortality of FSNC is depicted in Fig. 6a. The results revealed that percent mortality of FSNC treated 1^st^, 2^nd^, 3^rd^, 4^th^ larval instars and pupae of *A. aegypti* is 100% with the film fabricated using 200 μl of the nanocomposite suspension. In case of FGNC, around 86–98% mortality was observed for all the larval stages and pupa against the film fabricated with 200 μl of nanocomposite suspension as shown in Fig. 6b. Typically, the weights of the resultant dry films (corresponding to 200 µl dispersion) were 11.36 ± 0.45 mg, 10.03 ± 0.41 mg and 9.6 ± 0.26 mg for FSNC, FGNC and PF, respectively. It is reported that the green synthesis of silver nanoparticles has the potential to be used as an eco-friendly approach to control the mosquitoes^56–58^. With larvicidal bioassay study, it is important to investigate the toxicity of silver and gold nanoparticles in non-target species models. Hence, the toxicity of PF, FSNC and FGNC to the non-target species *P. reticulate* was detected under laboratory conditions. The results did not reveal any noticeable effects on *P. reticulate* even after 24 h and 48 h of exposure. It is reported that silver ions and stabilizing agents disclosed no significant defects in developing the zebrafish embryo^59^. In the present investigation, it has been found that silver nanoparticles possess more potential for mosquito control than that of gold nanoparticles. Similar results were recorded for fungus generated (*C. tropicum*, MTCC 2828) gold and silver nanoparticles against the larvae of *Culex quinquefascitus* and *Anopheles stephensi* ^60^. Silver and gold nanoparticles, like almost all nanoparticles, are potentially toxic beyond a certain concentration because the survival of the organism is compromised due to scores of pathophysiological abnormalities past that concentration. Nevertheless, biochemical analysis, suggests the interaction of nanoparticles with biomolecules. In this study, the larvae treated with FSNC exhibited decreased protein quantity as compared to control and FGNC treated group (Fig. 7). The same pattern was observed in case of DNA content; while RNA content of larvae was decreased when treated with FGNC and a slight decrease was observed in FSNC treated set as compared with control as displayed in Fig. 7. It was reported that the nanoparticles bind to functional groups of proteins which results in protein deactivation and denaturation^61,62^.

### Phytotoxicity study

**Figure 8:**
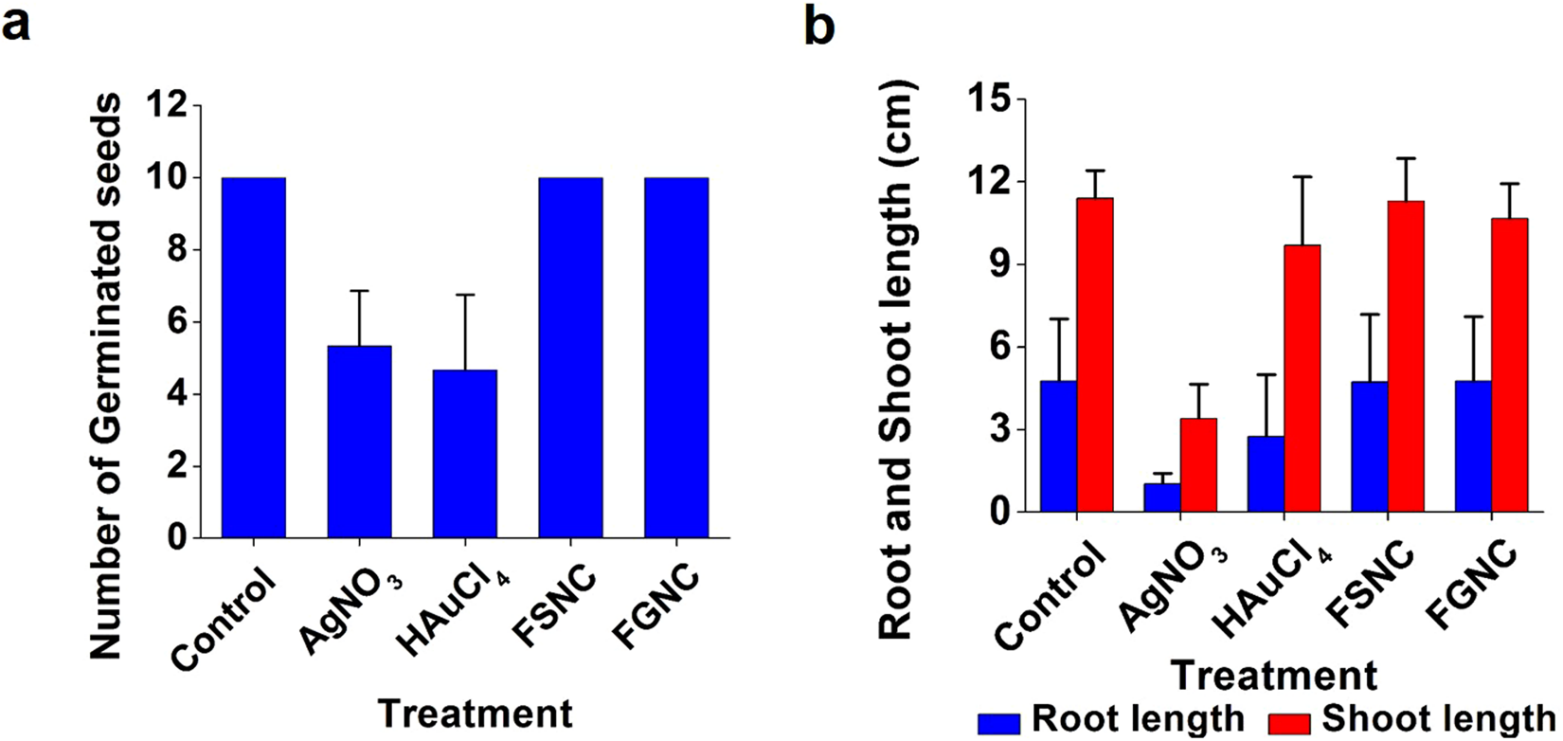
Effects of silver nitrate, auric chloride, FSNC and FGNC on (**a**) seed germination and (**b**) root & shoot length of *Triticum sativum* after seven days.

Germination is important for determining the density of the plants. Based on the reports of effects of nanoparticles on seed germination mechanisms, the nanoparticles increase water absorption by seeds and also enhance the activities of some enzymes and such changes improve the seed germination in some plants^63,64^. However, few studies reported that nanoparticles of silver inhibited biomass unlike the previous reports^65^. Thus, there are several controversies over the toxicity of nanoparticles on seed germination, biomass and root, shoot length. In the present research work, we proposed a novel method of nanoparticle immobilization, so that the nanoparticles cannot easily percolate the irrigated water. From Fig. 8a, it is clear that silver nitrate and auric tetrachloride dispersion have negative effects on seed germination of *Triticum sativum* as compared to control, FSNC and FGNC group. The results indicated that FSNC and FGNC do not exert toxic effects on *Triticum sativum* seed germination. We also carried out study on the effects of silver nitrate, auric chloride, FSNC and FGNC films on root-shoot length of *Triticum sativum*. The results, as shown in Fig. 8b, indicate that the FSNC does not reveal any adverse effects on root and shoot length as compared to control while FGNC exhibits slight decrease in shoot length. It was also observed that silver nitrate hampers the growth of root and shoot as compared to auric chloride as shown in Fig. 8b.

**Figure 9:**
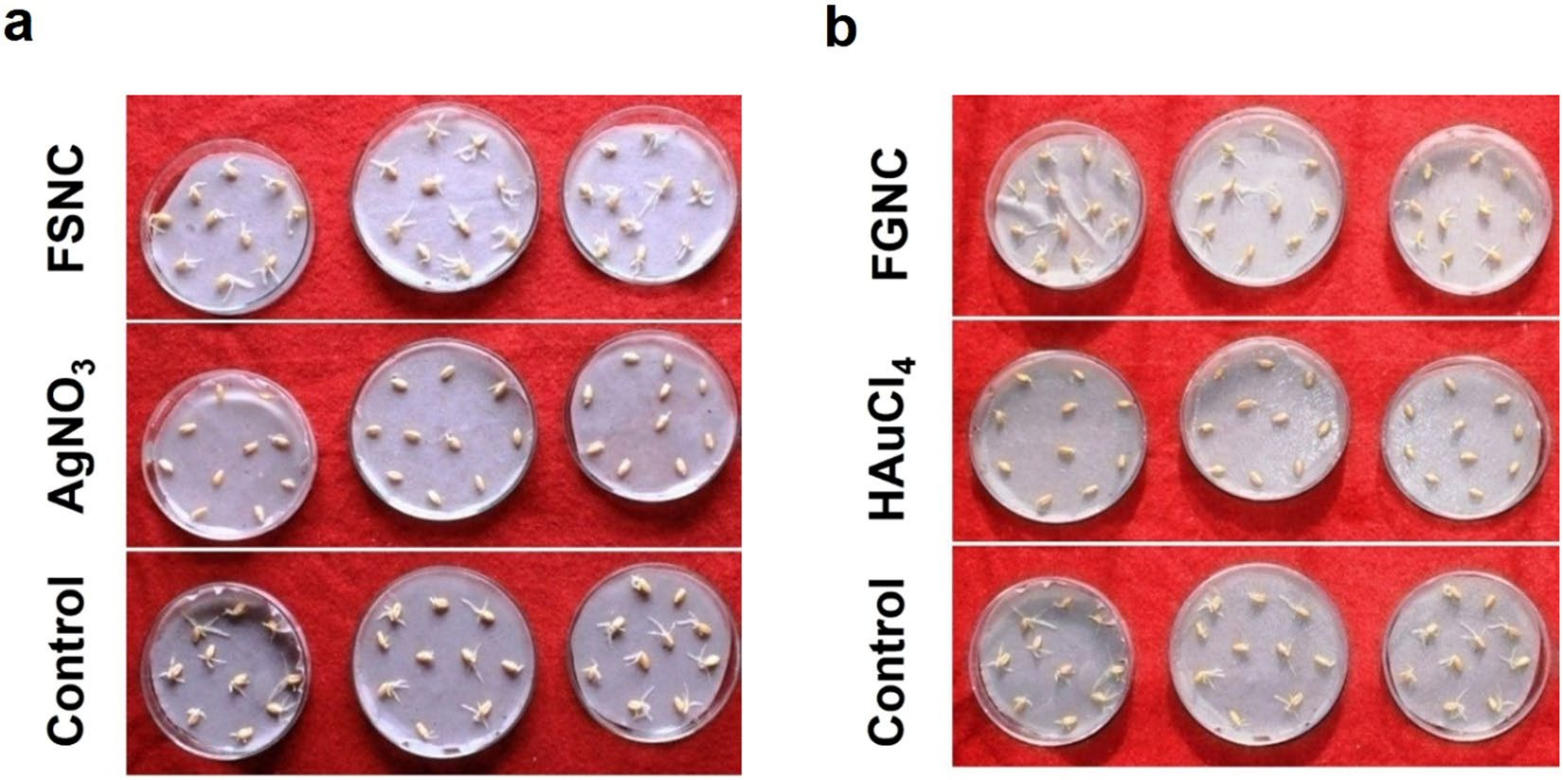
Effects of (**a**) FSNC and (**b**) FGNC on seed germination of *Triticum sativum*.

The digital photographs of the germinated seeds when treated with pure silk fibroin films (control), silver nitrate and FSNC films (Fig. 9a) and auric tetrachloride and FGNC films (Fig. 9b) confirm the aforesaid results pertaining to seed germination. The present phytotoxicity study is very significant as it asserts that use of bionanocomposite films (with effective surface immobilization of oligodynamic nanoparticles in the biocompatible SF matrix) can be a safer alternative as bio-chemical agent in performing larvicidal action even in big water bodies like water tanks and storage units. The resultant water can be rendered as safe for aquatic as well as for plant life. This is presumably the first report on such kind of study.

**Figure 10:**
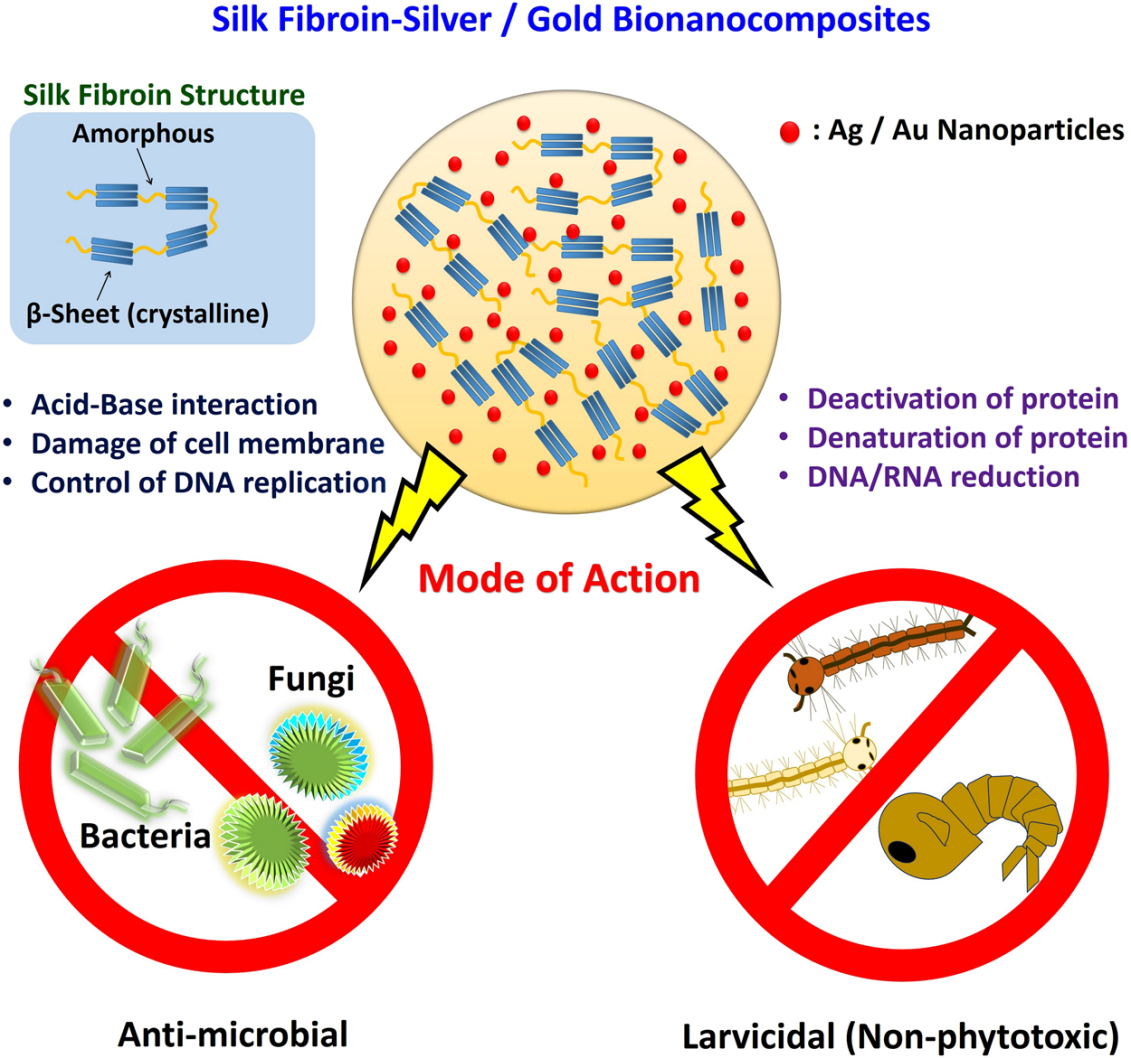
Schematic illustration of the antimicrobial and larvicidal action of the silk fibroin- Ag/Au bionanocomposites.

The overall schematic illustration of the speculative antimicrobial and larvicidal action path for the silk fibroin- Ag/Au bionanocomposite systems is provided in Fig. 10.

## Experimental

### Preparation of aqueous silk fibroin solution

The pure silk fibroin was obtained by following the standard protocol^32^. In brief, 5 grams of dried and cut pieces of *B. mori* silk cocoons were degummed by boiling in aqueous solution of 0.02 M sodium carbonate. While, fibroin solution was prepared by dissolving degummed silk in 9.3 M LiBr at 70 °C for 2 h. The fibroin solution was dialyzed in cellulose membrane based dialyzed cassette (Thermo Scientific, molecular weight 10000 KD) against deionized water for 3 days. The protein content of dialyzed fibroin was found to be around 35 mg/100 ml of the solution. This dialyzed fibroin was used in the synthesis of silver and gold nanocomposites.

### Synthesis of nanoparticles

The silver nanoparticles within silk fibroin solution were synthesized by dissolving 1 mM of silver nitrate (AgNO_3_) in 10 ml dialyzed fibroin. The flask was incubated at room temperature in natural sunlight till its color changed from colorless to brown. Gold nanoparticles were synthesized by dissolving 0.5 mM auric chloride (HAuClO_4_) in 10 ml of fibroin. The flask was incubated at room temperature in dark condition until its color changed to ruby red.

### Fabrication of FSNC and FGNC films

The films of fibroin containing silver and gold nanoparticles were fabricated by drying the silk fibroin dispersion containing metal nanoparticles by drop casting method. The liquid suspension of FSNC and FGNC was spread in a circular manner and allowed to dry in a laminar air flow. Post-drying, the fabricated films were passed through various grades of ethanol (10%, 25%, 50% and 100%) and finally washed with distilled water and allowed to dry at room temperature. The films of pure fibroin were also fabricated by the same procedure to act as control.

### Physico-chemical characterization of the films

The optical properties of the resultant nanocomposite dispersions and films were deduced from their absorbance spectra recorded with UV-Visible spectrophotometer (JASCO, V-670) in the range 400–800 nm. Surface topography of the films was examined with atomic force microscopy by Scanning Probe Microscope (JSPM-5200, JEOL).

### Antimicrobial activity, MIC and MBC/MFC of FSNC and FGNC

The screening of antimicrobial activity of FSNC and FGNC dispersions were carried out by the agar well diffusion method using nutrient agar (NA) medium. The bacterial inocula were prepared from the colonies of 24 h culture on nutrient agar medium. The inoculum was adjusted to final concentration of approximately 10^6^ CFU/ml for the bacteria. The bionanocomposite films of the individual FSNC and FGNC dispersions and plane fibroin (PF) were fabricated using 50 µl of the respective dispersions. The weights of the resultant dry films were 2.79 ± 0.11 mg, 2.51 ± 0.11 mg and 2.35 ± 0.08 mg for FSNC, FGNC and PF, respectively. These film-samples were imbibed on the test media which were previously inoculated with each test strain. Plates were incubated at 37 °C and inhibition zones were measured after 24 h of incubation. Standard drug ciprofloxacin (10 µg/well) served as the positive antibacterial control^66^. MIC was determined by serially diluting the FSNC and FGNC dispersions in the concentration of 4, 6, 8 and 10 µg/ml. Microorganisms were grown in liquid medium consisting of Mueller Hinton broth at 37 °C, MIC represents the lowest concentration required to inhibit the growth of microorganisms. All assays were carried out for three times and the control test was carried out with the sterilized distilled water^67^. The MBC of the FSNC and FGNC dispersions was determined by taking samples from tubes with no visible growth in the MIC assay and sub-cultured on freshly prepared nutrient agar plates and later incubated at 37 °C for 48 h. The MBC was taken as the concentration of the FSNC and FGNC dispersions that did not show any growth on a new set of agar plates^68^.

### Determining the growth curves of bacterial cells

To examine the growth curves of bacterial cells, the bacterial cell concentration in Muller-Hinton broth was adjusted to 10^6^ CFU/ml and then exposed to FSNC and FGNC dispersions at different concentrations (1/2MIC, MIC and 2MIC). Each culture was incubated in a shaking incubator at 37 °C for 24 hours. Growth curves of bacterial cell cultures were attained through repeated measurements of the optical density (OD) at 600 nm.

### Mode of action of FSNC and FGNC on bacterial cells

The MIC of FSNC and FGNC was used to study the mode of action on bacteria, and the concentration of bacteria was adjusted to 10^6^ CFU/ml exposed to the nanocomposites separately for 6 hr. One ml of sample was withdrawn from each set and the concentration of protein reducing sugar, DNA and RNA was determined. The method of Heipieper^69^ was also followed to determine the leakage at UV260 and UV 280 absorbing material.

### Larvicidal bioassay

For the laboratory trial, *Aedes aegypti* was breed in the laboratory to obtain uniform age. Ten larvae (1^st^ to 4^th^ instar and pupae) were placed in dechlorinated water in the glass beaker and one film of FSNC and FGNC was kept separately and 0.5 mg larval food was provided for each test^70,71^. The larvicidal activity against each instar and the pupae were replicated thrice. In each case, the control comprised 10 larvae/pupae in distilled water. Control mortality was corrected by using Abbott’s formula and percentage mortality was calculated^72^. The exposed larvae/pupae were used for biochemical analysis to study the mechanism of action of FSNC and FGNC. To determine the toxicity of FSNC and FGNC, a non- target organism *P. reticulate* was selected to carry out the toxicity test.

### Biochemical analysis

The concentration of protein was determined by Lowry methods using Bovine Serum Albumin (BSA) as standard^73^, reducing sugar by Miller method^74^, the total free amino acids were estimated by the Ninhydrin method^75^ while DNA and RNA were estimated by diphenylamine and orcinol method^76^, respectively.

### Phytotoxicity study

Silver and gold ions were used to compare the toxicities of FSNC and FGNC.

To carry out phytotoxicity study, clean seeds of *Triticum sativum* were treated separately with 5 ml of tap water as control, 5 ml of 1 mM AgNO_3_ solution, 0.5 mM of HAuClO_4_ solution and 5 ml of FSNC and FGNC dispersion (after carrying out larvicidal activity). The treated seeds were then transferred on a filter paper in the petri dishes, (with 10 seeds per dish) and further subjected to incubation for seven days and subsequently placed in a growth chamber maintained at 25°C and 12 h light. Each set was prepared in three replicas.

## Conclusions

In summary, we have devised a novel, versatile and controlled strategy to restrain individual nanoscale silver and gold within silk fibroin matrix. Our approach was based on the application of silk fibroin-noble metal nanoparticles for killing pathogenic micro-organisms, larvae of *A. aegypti* in dispersion form as well as film form without leading to any environmental threats. The FSNC and FGNC demonstrated an efficient antimicrobial activity as well as larvicidal activity. As the resultant silver and gold nanoparticles were effectively immobilized within natural biopolymeric matrix, such nanoparticles cannot appreciably enter into the ecosystems (including freshwater bodies) as has been evinced by the phytotoxicity studies. Therefore, it is a rather simple green route to produce silver and gold nanoparticles within SF matrix and may have great potential for external antimicrobial therapeutics as well as large-scale larvicidal applications on-site.
